# Uptake of smoking cessation aids by smokers with a mental illness

**DOI:** 10.1007/s10865-016-9757-3

**Published:** 2016-06-29

**Authors:** Alexandra P. Metse, John Wiggers, Paula Wye, Richard Clancy, Lyndell Moore, Maree Adams, Maryanne Robinson, Jenny A. Bowman

**Affiliations:** 1 University of Newcastle, University Drive, Callaghan, NSW 2308 Australia; 2 Hunter Medical Research Institute, Lot 1 Kookaburra Circuit, New Lambton Heights, NSW 2305 Australia; 3 Hunter New England Population Health, Longworth Ave, Wallsend, NSW 2287 Australia; 4 Centre for Translational Neuroscience and Mental Health, Mater Hospital Cnr Edith and Platt Streets, Waratah, NSW 2298 Australia

**Keywords:** Tobacco use, Mental disorders, Treatment utilisation, Pharmacotherapy, Behavioural aids, Smoking cessation

## Abstract

Psychiatric inpatient settings represent an opportunity to initiate the provision of tobacco cessation care to smokers with a mental illness. This study describes the use of evidence-based smoking cessation aids proactively and universally offered to a population of psychiatric inpatients upon discharge, and explores factors associated with their uptake. Data derived from the conduct of a randomised controlled trial were analysed in terms of the proportion of participants (*N* = 378) that utilised cessation aids including project delivered telephone smoking cessation counselling and nicotine replacement therapy (NRT), and Quitline support. Factors associated with uptake of cessation aids were explored using multivariable logistic regression analyses. A large proportion of smokers utilised project delivered cessation counselling calls (89 %) and NRT (79 %), while 11 % used the Quitline. The majority accepted more than seven project delivered telephone cessation counselling calls (52 %), and reported NRT use during more than half of their accepted calls (70 %). Older age, higher nicotine dependence, irregular smoking and seeing oneself as a non-smoker were associated with uptake of behavioural cessation aids. Higher nicotine dependence was similarly associated with use of pharmacological aids, as was NRT use whilst an inpatient. Most smokers with a mental illness took up a proactive offer of aids to support their stopping smoking. Consideration by service providers of factors associated with uptake may increase further the proportion of such smokers who use evidence-based cessation aids and consequently quit smoking successfully.

Tobacco smoking is one of the leading risk factors for preventable chronic disease and death in Australia and worldwide (Lim et al., 2012). Over the past two decades, the proportion of smokers in Australia (Australian Institute of Health and Welfare, 2011) and other high income countries (Cook et al., 2014; Office for National Statistics, 2014) has approximately halved. However among persons with a mental illness, smoking prevalence remains between 34 and 88 % (Cooper et al., 2012; de Leon & Diaz, 2005; McClave et al., 2010), with some of the highest rates of smoking observed among clients with psychotic disorders (de Leon & Diaz, 2005) and those within acute psychiatric settings (de Leon & Diaz, 2005; Stockings et al., 2013). People with a mental illness also smoke a greater number of cigarettes and are more nicotine dependent than smokers without a mental illness (Bowden et al., 2011; Lasser et al., 2000).

A reduced life expectancy of between 14 and 25 years is experienced by people with a mental illness; with tobacco related conditions, including cardiovascular disease and cancer, being the leading causes of premature mortality (Colton & Manderscheid, 2006; Lawrence et al., 2013). Historically smoking has been assumed to offer a means of managing mental health symptoms for this subgroup (de Leon et al., 2006), however recent evidence suggests cessation actually improves such symptoms (Prochaska, 2014; Taylor et al., 2014). The need to reduce the disproportionately high rate of smoking among persons with a mental illness has been recognised internationally as a public health priority (Lawrence et al., 2009; Royal College of Physicians & Royal College of Psychiatrists, 2013).

Despite evidence suggesting persons with a mental illness have a desire to quit (Stockings et al., 2013; Szatkowski & McNeill, 2015), attempt to quit at rates similar to that of smokers generally (McClave et al., 2010), and that quitting aids [e.g. telephone counselling, nicotine replacement therapy (NRT)] are efficacious for this population (Banham & Gilbody, 2010; Gierisch et al., 2012), lower rates of quitting success are reported for smokers with a mental illness (George et al., 2000; Lasser et al., 2000; McClave et al., 2010; Stockings et al., 2013). Increasing access to and use of smoking cessation aids shown to be most effective in promoting quitting behaviours and cessation—multimodal approaches incorporating both pharmacotherapeutic and behavioural components (Banham & Gilbody, 2010; Fiore et al., 2008; Royal College of Physicians & Royal College of Psychiatrists, 2013)—is therefore of particular importance for smokers with a mental illness.

Offering cessation aids in a manner which is *proactive* and *universal*—that is, unsolicited, and irrespective of clinical characteristics (such as physical or mental health status) or smoking characteristics such as ‘readiness to change’)—has been reported to increase the uptake of such aids among smokers in the general population. For example, telephone based proactive and universal recruitment of smokers to Quitline cessation services has been reported to result in 52 % of smokers using such a service (Tzelepis et al., 2009); a greater proportion than the 4 % of smokers who contact the service on a self-referral basis (Miller et al., 2003). Notably, the smoking cessation outcomes achieved by smokers recruited in this way (Tzelepis et al., 2011) appear to equate to those for smokers who use Quitlines on a self-referral basis (Zhu et al., 2002).

Review evidence suggests admission to general (Rigotti et al., 2012) and psychiatric (Royal College of Physicians & Royal College of Psychiatrists, 2013) hospitals provides a key opportunity to initiate smoking cessation care, particularly since the introduction of smoke-free policies in such settings. With respect to such care offered immediately following discharge from a psychiatric inpatient facility, only two studies have reported the uptake of *proactively* and *universally* offered smoking cessation aids (Schuck et al., 2014; Stockings et al., 2014). Stockings et al. (2014) reported 90 and 68 % of participants accepted an offer of telephone counselling and NRT respectively on at least one occasion post discharge. Approximately 50 % of such participants utilised seven or more weeks of counselling and/or NRT. No analysis was reported regarding the factors associated with uptake of smoking cessations aids. In the second study, Schuck et al. (2014) reported 88 % of participants opted to receive a 1 month supply of NRT following discharge from an inpatient psychiatric facility, with approximately half of those requesting a second months supply. Later stage of change, more severe mental health symptoms and nicotine dependence, female gender and older age were associated with uptake of the initial offer of NRT, whilst older participants and those with more severe mental health symptoms were more likely to request a subsequent supply. No study has as yet explored the factors associated with uptake of both pharmacotherapeutic and behavioural aids when offered to all smokers following a psychiatric admission. Identifying such factors may inform strategies to encourage the use of efficacious multimodal cessation supports.

This study describes the uptake of NRT and two telephone based behavioural aids (project delivered telephone smoking cessation counselling and Quitline), proactively offered to a population of Australian mental health patients upon discharge from an inpatient facility, and explores factors associated with their uptake. On the basis of limited previous research (Schuck et al., 2014), smokers who are: older, female, contemplating quitting, more nicotine dependent, and experiencing more severe psychiatric illness may be more likely to take up NRT.

## Method

### Design and setting

A descriptive study was undertaken of patients following discharge from four acute adult psychiatric inpatient facilities in New South Wales (NSW), Australia.

### Sample and recruitment procedure

Between October 2012 and April 2014, research staff approached all admitted patients in the four facilities to assess their eligibility for participation in a randomised controlled trial assessing the efficacy of providing smoking cessation support upon discharge. Eligible patients were: current smokers (smoked tobacco in the month prior to admission), 18 years of age or above, willing to provide contact details to facilitate communication post discharge, and able to give informed consent. Consenting patients completed a baseline interview and were the allocated to either a usual care or intervention condition (Metse et al., 2014). Those in the intervention condition constituted the sample for this study.

### Intervention delivery post discharge

Once discharged from hospital, participants were proactively offered: 12 weeks of fully subsidised NRT, 16 weeks of project delivered telephone smoking cessation counselling, and a proactive referral to the NSW Quitline telephone service. Each participant was allocated to a counsellor trained in motivational interviewing (Miller & Rollnick, 2002) who provided all components of the intervention (telephone cessation counselling, NRT provision, and proactive Quitline referral). Between 11 and 15 telephone cessation counselling calls were provided over a 16 weeks period (the first within 3 days of discharge, then weekly for 5 weeks, weekly or fortnightly tailored in accordance with participant preference for the subsequent 7 weeks, and fortnightly between weeks 12 and 16). When calls were unanswered counsellors reattempted daily until the due date of the next call.

NRT was offered to participants during every cessation counselling call in the first 12 weeks of the intervention. If the offer of NRT was accepted, it was posted to participants immediately following the completed cessation counselling call. Participants were offered and encouraged to utilise a combination of both patches and adjunctive forms of NRT, the latter of which could include lozenges, mini lozenges, gum, inhalers, and oral spray.

The counselling staff offered participants a proactive Quitline referral at the time of the first telephone call. The NSW Quitline offers a free and individually tailored telephone service to assist consumers in the process of quitting. This service commonly offers advice and support surrounding quitting preparation, relapse prevention, and cessation maintenance.

### Data collection procedures

Clinical and demographic information was obtained via the electronic medical record system. Characteristics of patient smoking behaviours, social and environmental factors, and other demographic data were collected for all participants via face-to-face interview during the inpatient stay.

Smoking variables in hospital were collected for a subsample of participants by research staff delivering the telephone cessation counselling during the first call post discharge. Data pertaining to uptake of proactively offered smoking cessation aids were collected by participant self-report (use of NRT and Quitline) and logs of intervention delivery (number and duration of calls) completed by telephone counsellors at the time of each call.

### Measures

#### Uptake of smoking cessation aids

Use of project delivered cessation counselling: the measures were contact status (accepted at least one counselling call [yes, no]), and number and duration of calls accepted. A call was recorded as ‘accepted’ if the following was achieved: smoking status and NRT requirements assessed, and at least one (of 16) motivational interviewing techniques implemented. Examples of such techniques include exploring importance and confidence to change, and rolling with resistance.

Use of NRT: the measures were self-reported use of project supplied NRT throughout intervention (yes, no), type of NRT used (patch only, adjunctive only, patch and adjunctive), and number of calls reporting NRT use.

Use of Quitline: a single measure assessed participant self-reported use of Quitline at any point during intervention period (yes, no).

#### Clinical and demographic features

Information collected via the patient medical record system included: age, gender, relationship status (single, married/de facto, separated/divorced, widowed, did not state/inadequately described), Aboriginal and/or Torres Strait Islander status (Aboriginal and/or Torres Strait Islander, neither or unknown), mental health diagnosis at the time of discharge (schizophrenia and related psychosis, anxiety and stress related disorders, mood disorders, substance-related disorders, personality and other disorders), legal status on admission (voluntary, involuntary), and total length of stay (total days between admission and discharge including periods of leave).

Information collected as part of the face-to-face interview included: education (primary school, third year of high school, school certificate, Higher School Certificate [HSC], TAFE certificate or diploma, bachelor degree, post graduate degree), employment details (full time, part time, household duties, student, unemployed/other), receipt of a government payment (yes, no), and level of alcohol use (AUDIT-C) (Bradley et al., 2007).

#### Characteristics of smoking

Measures at baseline were: smoking status (daily smoker, weekly smoker, irregular smoker [smoked cigarettes less than weekly in month prior to admission]), cigarettes per day, level of nicotine dependence (Fagerstrom Test for Nicotine Dependence [FTND]) (Heatherton et al., 1991), age when started smoking, number of years smoked, readiness to quit (Readiness to Quit Questionnaire) (Crittenden et al., 1994), identity as a smoker (ease of seeing self as a non-smoker [easy, difficult, unsure]) (West, 2006), and quitting history (ever tried to quit [yes, no]; quit attempt in past 12 months [yes, no]).

#### Social and environmental factors

Measures at baseline were: lived in a smoke free house prior to admission (smoke free house was defined as a place of residence where no one is allowed to smoke inside; yes/no), lived with smokers prior to admission (yes, no), and current support from anyone in their life to quit smoking (yes, no).

#### Smoking variables in hospital

Measures collected at the time of the first supportive telephone call post discharge were: participant use of NRT (yes, no) and smoking behaviour (smoked, did not smoke) whilst in hospital. These measures were introduced after the commencement of data collection, and are available for the latter 45 % of participants recruited.

### Analysis

Data were analysed using IBM SPSS Statistics version 22.

The following variables were transformed from numerical to categorical for the purpose of the association analysis: age when started smoking (≤14 years, >14 years) (Siahpush et al., 2003), number of years smoked (≤10, >10–≤20, >20 years) (Khuder et al., 1999), and alcohol use (AUDIT score of ≥3 for women and ≥4 for men was considered to be harmful/hazardous, scores below these cut offs were coded as non-harmful/hazardous) (Bradley et al., 2007).

The following categorical variables were reduced to two levels: diagnosis (psychotic, non-psychotic disorders) (de Leon & Diaz, 2005; George et al., 2000), relationship status (partnered, not partnered), employment (paid employment, no paid employment), smoking status (daily smoker, weekly/irregular smoker), readiness to quit (pre-contemplative, contemplative or a more progressed stage) (Crittenden et al., 1994), ease of seeing self as non-smoker (easy, difficult/unsure), type of NRT used during the intervention (single, optimal NRT use [patch plus adjunctive]) (Stead et al., 2012). Highest level of education attained was reduced to three levels (up to school certificate, HSC, tertiary).

Descriptive statistics were used to summarise patient clinical and demographic characteristics, characteristics of smoking, social and environmental factors, smoking variables in hospital, and the degree of intervention uptake. Categorical data were described using proportions and continuous data using means, standard deviations, medians and ranges.

Chi square and logistic regression analyses were used for exploratory analysis of possible univariate associations between measures of cessation aid uptake and a range of variables: social and demographic features, characteristics of smoking (including motivation to quit), social and environmental factors, and smoking variables in hospital (included in Tables 1 and 2). Univariate associations with a *p* value of ≤0.25 were entered into multivariable logistic regression models (Bursac et al., 2008) using both backward elimination and stepwise variable selection to determine model stability. If applicable, generalised linear mixed models (GLMMs) were used to account for over dispersion by adding a random effect to account for variability between participants. Six logistic regression models were developed: acceptance of at least one project delivered cessation counselling call (yes, no); proportion of project delivered calls accepted (*n*/15); use of NRT (yes, no); proportion of calls reporting NRT use (*n*/number calls accepted); type of NRT used (single form, patch plus adjunctive); use of Quitline (yes, no). The critical *p* value was set at *p* ≤ 0.01 to account for multiple comparisons.

**Table 1:** Clinical and demographic features of approached and not approached patients, and participants and non-consenters

	Not approached (*n* = 1311)	Approached (*n* = 2315)	Non-consenters (*n* = 483)	Study participants (*n* = 378)
Gender (%)				
Male	60.0	55.4	63.4	61.4
Female	40.0	44.6	36.6	38.6
Age (years)^2,5^				
Mean (SD)	39.8 (17.1)	41.8 (14.2)	38.9 (11.7)	39.1 (12.0)
Median (range Min–Max)	37 (10–94)	41 (18–93)	38 (18–82)	38.5 (19–74)
Relationship status (%)				
Single	59.0	58.6	70.8	66.4
Married/De facto	25.7	24.1	17.4	18.0
Separated/divorced	11.0	14.2	10.2	12.7
Widowed	3.2	2.3	0.6	2.4
Not stated/inadequately described	1.0	0.7	1.0	0.5
Aboriginal and/or Torres Strait Islander status (%)^1,2,3^				
Aboriginal and/or Torres Strait Islander	12.8	11.6	17.7	14.0
Neither Aboriginal or Torres Strait Islander/unknown	87.2	88.4	82.3	86.0
Diagnosis type (%)^2,3,4^				
Schizophrenia and related psychosis	14.1	27.6	37.1	19.0
Anxiety and stress related disorders	20.3	8.5	6.4	11.9
Mood disorders	23.1	30.8	22.4	27.0
Substance-related disorders	21.2	15.6	18.0	25.4
Personality and other disorders	21.3	17.4	16.1	16.7
Length of stay (days)				
Mean (SD)	12.4 (62.1)	16.8 (28.7)	17.6 (24.4)	15 (18.7)
Median (range Min–Max)	2 (0–1715)	10 (0–945)	10 (0–236)	8 (0–121)
Legal status on admission (%)^1,4,5^				
Voluntary	55.6	53.2	49.3	52.1
Involuntary	44.4	46.8	50.7	47.9
Employment status (%)^1^				
Full time	–	–	–	13.0
Part time	–	–	–	12.7
Student	–	–	–	2.6
Unemployed/household duties/other	–	–	–	71.7
Highest education level achieved (%)^1,2,3,4,6^				
Up to Third year of high school	–	–	–	28.8
School certificate	–	–	–	34.1
Higher school certificate (HSC)	–	–	–	15.1
Tertiary	–	–	–	22.0
Receipt of a government payment (%)^4,5,6^				
Yes	–	–	–	78.0
Alcohol use (AUDIT-C) (%)^a,1,2,5^				
Harmful/hazardous	–	–	–	64.5
Non-harmful/hazardous	–	–	–	35.5

Entered in to regression analyses: ^1^ Acceptance of at least one telephone supportive call; ^2^ Accepting a higher proportion of calls; ^3 ^NRT use; ^4^ Higher proportion of calls reporting NRT use; ^5^ Optimal use of NRT; ^6^ Use of Quitline

– Data not obtained for respective sample

^a^n = 377 due to missing data

**Table 2:** Characteristics of smoking, social and environmental factors, and smoking variables in hospital

	Total (*N* = 378)
Smoking status (%)^6^	
Daily	93.4
Weekly	3.4
Irregular	3.2
Cigarettes per day^1,2^	
Mean (SD)	21.8 (14.4)
Median (range Min–Max)	20.0 (1–100)
FTND total^1,2,4,5,6^	
Mean (SD)	5.5 (2.5)
Median (range Min–Max)	6.0 (0–10)
Readiness to quit (%)^3,6^	
Pre-contemplative	55.3
Contemplative or a more progressed stage	44.7
Age when started smoking (%)^4,5^	
<12	23.3
12–<14	21.2
14–<16	20.9
16–<18	17.5
18+	17.2
Number of years smoked (%)^2,5,6^	
≤10	16.4
>10–≤20	28.0
>20	55.6
Ever tried to quit (%)^1,2,3,6^	
Yes	87.0
Quit attempt in past 12 months (%)^2,4,5,6^	
Yes	49.2
Ease of seeing self as non-smoker (%)^1^	
Easy	40.2
Difficult	43.4
Unsure	16.4
Lived in a smoke-free house (%)^1,4^	
Yes	70.1
Lived with smokers (%)	
Yes	43.7
Support from anyone to quit smoking (%)^2,6^	
Yes	94.7
Smoked in hospital (%)^a,2,4^	
Yes	52.1
Used NRT in hospital (%)^a,3,4^	
Yes	85.2

Entered in to regression analyses: ^1^ Acceptance of at least one telephone supportive call; ^2^ Accepting a higher proportion of calls; ^3 ^NRT use; ^4 ^Higher proportion of calls reporting NRT use; ^5^ Optimal use of NRT; ^6 ^Use of Quitline

*FTND* Fagerstrom Test for Nicotine Dependence

^a^N = 169

## Results

### Sample

Of the 3626 patients admitted to the four inpatient facilities within the recruitment period, 64 % (*n* = 2315) were approached by research staff, with patients not being approached due to either a short length of stay (≤one night; 38 %) or psychiatric instability for the duration of time spent as an inpatient (35 %). Of the patients approached, 53 % (*n* = 1237) were eligible, of whom 61 % (*n* = 754) consented to participate in the trial. Three hundred and seventy-nine patients were allocated to receive the intervention. One participant allocated to the intervention condition was not discharged at the time of project completion, providing a sample for this study of 378 patients. Data pertaining to smoking care received in hospital were collected for 169 participants.

### Patient clinical and demographic features, characteristics of smoking, and smoking variables in hospital

Patients approached to participate in the trial, compared to those not approached, had a longer length of stay; and were more likely to have a mood disorder or be diagnosed with schizophrenia and related psychosis; and less likely to be diagnosed with an anxiety disorder. Study participants were less likely than non-consenters to be diagnosed with schizophrenia and related psychosis and more likely to be diagnosed with an anxiety disorder.

Table 1 describes the clinical and demographic features of patients approached/not approached and study participants/non-consenters. Table 2 describes participant smoking characteristics, social and environmental factors, and smoking variables in hospital.

### Uptake of Smoking Cessation Aids

#### Use of project delivered cessation counselling

The median time taken to initially contact participants post discharge was 3 days, with calls having a mean duration of 14.6 (*SD* = 11) minutes each. The large majority of participants (89 %) accepted at least one cessation counselling call, and the mean number of calls accepted was 7 (*SD* = 4.3) (Table 3). Approximately half of the participants (52 %) accepted more than seven cessation counselling calls.

**Table 3:** Uptake of smoking cessation aids

	Total (*N* = 378)
Acceptance of at least one project delivered cessation counselling call (%)	
Yes	88.6
Time till contact post discharge (days)	
Mean (SD)	12.4 (29.1)
Median (IQR)	3 (7)
Number of project delivered calls accepted (%)	
0–3	27.0
4–7	21.4
8–11	39.4
12–15	12.2
Duration of project delivered calls accepted (min)	
Mean (SD)	14.6 (10.7)
Median (IQR)	13 (13)
Used NRT at all during the course of the intervention (%)	
Yes	79.1
Of those who used NRT (n = 299), type of NRT used (%)	
Patch only	7.4
Adjunctive only	17.4
Optimal (patch and adjunctive)	75.3
Proportion of completed calls reported using NRT (%)	
0–25	24.1
26–49	6.1
50–74	20.4
75+	49.5
Use of Quitline (%)	
Yes	10.8

#### Use of NRT

Seventy-nine percent of participants used NRT at least once throughout the intervention, 70 % reported using NRT during more than half of their accepted calls. Three quarters of participants (75 %) who reported using NRT at all, used the optimal combination (patch plus adjunctive) for at least some part of the time.

#### Use of Quitline

Eleven per cent of participants used Quitline throughout the intervention period.

### Smoking cessation strategy uptake and associations with patient characteristics, social and environmental factors, and smoking variables in hospital

Variables with a *p* value of ≤0.25 in the Chi square analyses and initially included in the multivariable logistic regression models are identified in Tables 1 and 2. Between six and 12 variables were entered into each model, contingent on the dependent variable of interest. Significant findings and those approaching significance are shown in Table 4.

**Table 4:** Predicting smoking cessation aid uptake from patient characteristics, social and environmental factors, and smoking variables in hospital

Predictor	B	SE	OR	95 % CI		*p*
				Lower	Upper	
Acceptance of at least one project delivered cessation counselling call^a^						
See self as non-smoker						
Difficult	−0.77	0.39	0.46	0.22	0.99	0.05
Easy	1					
Proportion of cessation counselling calls accepted^a^						
Age						
1 year	0.022	0.01	1.02	1.01	1.04	<0.001
10 years			1.25	1.11	1.42	
Use of NRT^a,b^						
Used NRT in hospital						
Yes	1.21	0.60	3.35	1.04	10.81	0.04
No	1					
Proportion of calls where NRT use was reported^a^						
Nicotine dependence^e^						
1 point	0.045	0.02	1.05	1.01	1.08	0.01
3 points			1.14	1.03	1.28	
Optimal NRT use (patch plus adjunctive)^c,d^						
Nicotine dependence^e^						
1 point	0.184	0.05	1.20	1.08	1.34	0.001
3 points			1.74	1.26	2.39	
Use of Quitline^a^						
Smoking status						
Daily	−2.27	0.55	0.10	0.04	0.31	<.001
Weekly/Irregular	1					
Nicotine dependence^e^						
1 point	0.232	0.08	1.26	1.09	1.47	0.003
3 points			2.01	1.27	3.15	

^a^(Reference category: No)

^b^This model includes data pertaining to smoking related care received in hospital (*n* = 169)

^c^(Reference category: single type of NRT)

^d^This model only includes participants who used NRT (*n* = 299)

^e^FTND

#### Acceptance of at least one project delivered cessation counselling call

A trend approaching significance was found for seeing self as a smoker: participants who found it difficult to see themselves as a non-smoker were half as likely as those who could easily see themselves as non-smokers, to accept at least one cessation counselling call post discharge (odds ratio [*OR*]: 0.46, *p* = 0.05; Table 4).

#### Proportion of cessation counselling calls accepted

The proportion of calls accepted increased as age increased (*OR for 1* *year increase in age*: 1.02, *p* < 0.001); thus older participants were more likely to accept a greater proportion of calls.

#### NRT use

A trend approaching significance was found for use of NRT in hospital: participants who used NRT whilst they were an inpatient were more than three times as likely to use NRT following discharge (*OR*: 3.35, *p* = 0.04).

#### Proportion of calls where NRT use was reported

The proportion of calls using NRT increased with increasing nicotine dependence (*OR for 1 point increase in nicotine dependence*: 1.05, *p* = 0.01), therefore those with higher nicotine dependence reported using NRT during a greater proportion of accepted calls.

#### Optimal NRT use

Nicotine dependence was associated with optimal NRT use: the likelihood of using both patches and adjunctive NRT increased with increasing nicotine dependence (*OR for 1 point increase in nicotine dependence*: 1.20, *p* = 0.001).

#### Use of Quitline

Baseline smoking status and nicotine dependence were associated with the use of Quitline. Compared to weekly/irregular smokers, daily smokers were 0.10 times less likely to have used Quitline throughout the intervention period. The likelihood of using Quitline increased as nicotine dependence increased (*OR for 1 point increase in nicotine dependence*: 1.26, *p* = 0.003).

## Discussion

This is the first study to describe the uptake of smoking cessation aids offered proactively and universally to a population of smokers upon discharge from an acute psychiatric inpatient facility, and to explore factors associated with the uptake of both pharmacological and behavioural aids. The study illustrated a high likelihood of use of cessation aids: almost all participants (89 %) utilised at least one cessation strategy and more than three quarters (79 %) used both pharmacological and behavioural aids. A higher level of nicotine dependence was found to be positively associated with the proportion of calls where NRT use was reported, optimal NRT use and use of Quitline. Greater use of behavioural aids (project-delivered telephone support calls and Quitline) was also associated with older age and other smoking-related characteristics; demonstrating some concordance with findings of previous research among both smokers with a mental illness (Schuck et al., 2014) and in the general population (Tzelepis et al., 2012), and suggesting that interventions might be tailored to such characteristics in order to promote uptake. A trend approaching significance was found for a positive association of NRT use during the inpatient stay with a greater likelihood of uptake of NRT offered post discharge; although this finding was not a strong association, it may have particular clinical and policy significance and is worthy of further exploration. The study suggests that proactive and universal offers of cessation aids by clinicians, and consideration of factors associated with uptake, could increase the proportion of smokers with mental illness who use evidence-based cessation aids.

The rate of uptake of pharmacological and behavioural smoking cessation aids was comparable to other studies that proactively and universally offered cessation supports to persons with mental illness (Schuck et al., 2014; Stockings et al., 2014) and general population smokers (Fiore et al., 2004; Murray et al., 2013; Tzelepis et al., 2009). Notably the rate of uptake of NRT in this study was approximately one and a half times that reported in a similar trial where NRT was selectively offered only to smokers who were ‘ready to quit’, upon discharge from a psychiatric inpatient facility (Prochaska et al., 2014). These findings support research among general population smokers regarding the benefits of universally offering cessation aids to all smokers, including those who may not indicate being ready to quit at that time (Carpenter et al., 2004; Rigotti et al., 2012; Tzelepis et al., 2009).

In terms of the use of behavioural smoking cessation aids, the great majority of participants (89 %) accepted at least one project delivered telephone cessation counselling call, with the likelihood of doing so perhaps increased by being able to see oneself as a non-smoker, although this association was a trend only. Strength of smoking identity has also been suggested to be associated with quitting-related motivations or behaviours (Stockings et al., 2013; van den Putte et al., 2009). Among general population smokers, van den Putte et al. (2009) found weak identification as a smoker to be associated with increased quit attempts and Stockings et al. (2013) found, among mental health inpatients, that lack of enjoyment from smoking was associated with readiness to quit. Such findings together with those of the present study suggest smoking identity may be a psychological barrier to both smoking cessation and utilising cessation aids, and potentially of value to consider in the design of interventions which offer aids to smokers with mental illness. Further, the finding that a greater proportion of calls were accepted by older rather than younger participants is congruent with young adult smokers in general being more difficult to engage in smoking cessation intervention (Villanti et al., 2010), and with research among smokers with a mental illness suggesting that older persons are more likely to use smoking cessation medications consistently throughout an intervention (Schuck et al., 2014).

Approximately 10 % of participants used Quitline; at least double the proportion of smokers in Australia who might be expected to contact the service annually on a self-referral basis (Miller et al., 2003). This rate of uptake is especially notable given that a proactive Quitline referral was systematically offered by project counsellors on only a single occasion, at the point of first contact post discharge, and further that it occurred in the context of the intensive telephone based behavioural support already being offered by project counsellors. The likelihood of using Quitline increased with increasing nicotine dependence, a finding congruent with previous research that found higher nicotine dependence to increase the likelihood of taking up smoking cessation intervention among persons with a mental illness (Schuck et al., 2014). By contrast, participants who smoked daily prior to hospital admission, as opposed to less often, were less likely to utilise the Quitline, perhaps being more likely to see the intensive phone support offered by the project as more suitable to their needs. Such a suggestion remains speculative however and other explanations may also be posited.

With respect to the uptake of pharmacological smoking cessation aids, a substantial majority (79 %) of participants used NRT on at least one occasion. An association approaching significance suggested that the likelihood of doing so may have been increased by the use of NRT during the inpatient stay. Previous research has suggested that suboptimal and selective provision of nicotine dependence treatment within smoke-free psychiatric inpatient settings increases the likelihood of patients experiencing nicotine withdrawal symptoms (Prochaska et al., 2004), and the findings of this study suggest that research should further explore whether it may also decrease the proportion of patients who utilise NRT for cessation support following discharge. Seventy per cent of participants used NRT during ≥50 % of their accepted calls, and 75 % of those who used NRT post discharge used it ‘optimally’ for at least some part of the time, that is, using a combination of both patch and adjunctive forms of NRT (Stead et al., 2012). Participants with higher nicotine dependence were more likely to report NRT use during a higher proportion of calls and use NRT optimally; associations with evident clinical congruency.

Neither psychiatric diagnosis nor a measure of motivation (readiness to quit) were associated with any measure of strategy uptake. The former finding suggests positively that such aids may have equal appeal regardless of the nature or severity of psychiatric illness, and the latter that patients can be engaged in using cessation supports even if not assessed initially as having a high motivation to quit. Both findings suggest support for the benefit of proactively and universally offering smoking cessation aids to smokers with a mental illness.

The findings of the current study should be considered in the context of a number of its design characteristics. Patients who stayed in the hospital for one night or less and those with psychotic type disorders were underrepresented, while those with anxiety/stress disorders were over represented as compared to the facility’s entire patient population during the recruiting period. The study sample consisted of smokers who had consented to participate in a smoking related trial; and although ‘readiness to quit’ was not an eligibility criterion, the outcomes of this study may have been influenced by a self-selection bias. In addition, the possible impact on strategy uptake of a number of different counsellors occasionally delivering the intervention to particular participants, and infrequent disruption of intervention delivery as a result of circumstances such as participant readmission to hospital, could not be determined. Finally, while exploring the possible associations between the uptake of aids and smoking cessation was not within the scope of this paper, this will be addressed in a further publication.

The results from this study suggest that following an inpatient admission, a high proportion of smokers with a mental illness will take up proactively and universally offered cessation aids. Consideration of factors associated with uptake may further increase the proportion of smokers with mental illness who use evidence-based cessation supports, and consequently quit smoking successfully (Banham & Gilbody, 2010; Fiore et al., 2008). The provision of proactive Quitline referrals to all smokers upon discharge, may result in at least a two fold increase in the proportion of smokers utilising this service. Clinicians can therefore assist in redressing the significant smoking related health inequities experienced by persons with mental illness by following clinical guidelines (Fiore et al., 2008; New South Wales Department of Health, 2002) and proactively and universally offering patients smoking related care.
